# A multimodal approach to postoperative analgesia in ICU following major surgery

**DOI:** 10.1186/s42077-020-0052-8

**Published:** 2020-02-07

**Authors:** Sarah Vitug, Elizabeth Hong, Peter Roffey, Duraiyah Thangathurai

**Affiliations:** 1 The University of Queensland School of Medicine, Ochsner Clinical School, 1514 Jefferson Highway, New Orleans, LA 70121 USA; 2 Department of Anesthesiology, Keck Medical Center of the University of Southern California, 1500 San Pablo St, Los Angeles, CA 90033 USA

To the Editor,

Postoperative analgesia for complicated cardiovascular and oncologic surgical patients requires appropriate multimodal techniques in the intensive care unit (ICU). As the opioid epidemic continues to be problematic for physicians and patients, effective pain management techniques with an opioid-sparing effect are desired. Multimodal techniques afford significant pain relief; reduces opioid requirement, consumption, and related adverse effects; and expedites bowel recovery, earlier extubation, and overall shorter hospital stay (Mikhail and Thangathurai 1992; Aitkenhead 1989). Therefore, a combination of low-dose analgesics should be used and tailored to the patient’s clinical needs and nature of surgery.

For the past 3 years, our postoperative pain management involves ketamine (0.05–0.3 mg/kg/h) and fentanyl (1 μg/kg/h). To supplement, we add low dose dexmedetomidine (0.5 mg/kg/h), propofol (0.3 to 3 mg/kg/h), midazolam (0.05–2 mg/kg/h), acetaminophen (15 mg/kg every 4 h), or gabapentin (2 mg/kg up to do doses in 24 h). Regional techniques such as continuous lidocaine infusions (1.5–3 mg/kg/h), ON-Q pump (0.5% ropivacaine via epidural infusion 0.32 mg/kg/h), and TAP blocks (2.5–3 mg/kg ropivacaine) are considered in non-anticoagulated surgical patients. Most often, these patients may already have an existing epidural, and local anesthetic or opioids may be administered.

The benefits of an opioid-sparing approach are improved gastrointestinal function, minimal respiratory depression, mild sedation, and less opioid addiction. In turn, we observed early mobilization, fewer ventilator-dependent days (average reduction by 20%), and shorter hospital stay by 1–2 days. Of note, approximately 60% of patients suffered from chronic pain and were treated with a separate pain regimen. ICU patients were regularly monitored for signs of neurologic decline and blood pressure disturbances.

Ketamine has many therapeutic benefits such as hemodynamic stability, bronchodilation, minimal respiratory depression, analgesia, and amnesia. Ketamine also helps in acute and resistant-depressive states, which are common in the ICU. The rate of hallucinations and nightmares is < 5%, which are almost completely attenuated when ketamine is combined with midazolam or fentanyl (Hirota and Lambert 2018).

Sedative drugs such as propofol and dexmedetomidine may supplement patients who are on ventilators. Dexmedetomidine is generally reserved for hemodynamically stable patients, as bradycardia and hypotension may ensue (Shehabi et al. 2019). Intravenous acetaminophen can be used as an adjunct for pain in patients who have adequate liver function and no contraindications for hepatotoxicity. Gabapentin may be added for chronic pain. With this multimodal approach, we found a reduction in ICU and ventilator-dependent days, incidence of infections, and psychological issues such as depression and drug dependence.

In conclusion, postoperative pain control is best achieved with a combination of low-dose analgesics as opposed to any agent in isolation, as a single-medication approach is associated with more side effects, tolerance, and dependence. With multimodal techniques, specifically ketamine, fentanyl, and low-dose adjunct agents, we found that the overall opioid-sparing effect contributes to a reduction in ventilator-dependent days by up to 20%, faster bowel recovery and thus shorter hospital stay by 1–2 days, minimal vital organ dysfunction, and fewer psychological issues. Thus, patient wellness is optimized in the ICU with a multimodal approach to postoperative pain tailored to the patient’s needs.

## Data Availability

Not applicable

## Abbreviations

ICU: Intensive care unit
TAP: Transversus abdominis plane
